# Intersections of Modifiable Risks: Loneliness is Associated with Poor Subjective Sleep Quality in Older Women at Risk for Alzheimer's Disease

**DOI:** 10.1177/00914150241255888

**Published:** 2024-07-26

**Authors:** Madina Danish, Melanie A. Dratva, Kitty K. Lui, Nadine Heyworth, Xin Wang, Atul Malhotra, Sheri J. Hartman, Ellen E. Lee, Erin E. Sundermann, Sarah J. Banks

**Affiliations:** 1 MADURA ADAR Program, 8784 University of California, San Diego, La Jolla, CA, USA; 2 Department of Neurosciences, 8784 University of California, San Diego, La Jolla, CA, USA; 3 Joint Doctoral Program in Clinical Psychology, SDSU/UC San Diego, San Diego, CA, USA; 4 Department of Medicine, 8784 University of California, San Diego, La Jolla, CA, USA; 5 Department of Family Medicine and Public Health, 8784 University of California, San Diego, La Jolla, CA, USA; 6 Department of Psychiatry, 8784 University of California, San Diego, La Jolla, CA, USA

**Keywords:** Alzheimer's diseases, modifiable risk factors, loneliness, sleep quality, ADAR

## Abstract

We examined the relationship between subjective and objective sleep outcomes and loneliness in older women at risk for Alzheimer's disease (AD). Our sample consisted of 39 participants (aged 65+) with mild cognitive deficits who completed the UCLA Loneliness Scale, the Pittsburgh Sleep Quality Index (PSQI), and an at home sleep test, to determine presence of obstructive sleep apnea. Based on sleep quality scores, individuals categorized as “poor sleepers” had significantly higher loneliness scores than “good sleepers.” However, total loneliness scores did not significantly differ between groups with or without sleep apnea. We found that higher loneliness was significantly associated to lower habitual sleep efficiency and sleep duration and was also influenced by use of sleep medication. Our findings suggest that increased loneliness relates to worse subjective sleep quality, but not to sleep apnea. These findings suggest that combined interventions targeting loneliness and sleep quality may be important for older women.

Alzheimer's disease (AD) is a progressive neurodegenerative disorder characterized by pathophysiological changes including the aggregation of amyloid beta and phosphorylated tau proteins resulting in a progressive decline in memory, cognition, and everyday functioning. AD gradually advances over time and can progress from subtle memory lapses and confusion in the early stages to changes in personality, severe impairments in global cognition, and even difficulties with recognizing family members, in the later stages (“Alzheimer's Disease,” n.d.).

In 2023, an estimated 6.7 million Americans aged 65 and older are living with AD. Notably, two-thirds of Americans diagnosed with AD are women (“Alzheimer's Disease Facts and Figures,” 2023). Growing evidence indicates that women exhibit a more aggressive profile of AD than men, which is characterized by greater pathological tau burden and steeper cognitive decline prior to an AD dementia diagnosis. Due to the prominent disease burden of AD and the lack of effective treatments, there is growing interest in improving modifiable risk factors for AD, as a means of reducing disease risk. Modifiable risk factors that can influence the risk of developing AD include smoking, obesity, poor sleep, social isolation, sedentary behavior, diabetes, hypertension, and hearing impairment (Livingston et al., 2020; Macedo et al., 2017). Sex differences have been found in the level of risk conferred for many of these risk factors (Nianogo et al., 2022). Given these sex differences and the increased prevalence of AD in women, it is important to characterize modifiable risk factors for AD within women.

As previously mentioned, sleep has been identified as a promising risk factor, given that it plays an important role in maintaining overall health and well-being. Quality sleep contributes to cognition by improving attention and memory (Siddarth et al., 2021), supporting the immune system, balancing hormones, and having positive mental health benefits (Ali et al., 2013; Alvaro et al., 2013). Sleep also aids with brain detoxification, as during deep sleep, the glymphatic system becomes most active and clears out waste products from the brain, including the removal of amyloid beta and tau proteins (Reddy & van der Werf, 2020). These findings underscore sleep disturbances as an important modifiable risk factor for AD and highlight how sleep health may contribute to the prevention of AD.

Sleep disturbances become more common in older age and are characterized by an increased frequency of awakenings, a reduction in deep sleep, and a decrease in sleep duration (Li et al., 2018). These sleep problems, in addition to the reversal of day-night sleep patterns (extreme sleepiness during the day and insomnia at night), are significantly more common in older adults living with AD (Rose & Lorenz, 2010). In women without AD, studies have found worse sleep quality, longer sleep latency, shorter sleep duration, and a 40% greater likelihood of developing insomnia compared to men (Mallampalli & Carter, 2014). For women, an increase in susceptibility to developing sleep issues may be partially due to the effects of hormones and the menstrual cycle, which can trigger fluctuations in sleep quality and duration (Harrington et al., 2022). These sleep problems are further exacerbated for women during menopause with sleepdisrupting hot flashes (Freedman & Roehrs, 2007). Additional studies have shown that 40% to 70% of women in perimenopause report sleep problems and 20% to 30% continue to experience sleep disturbances after menopause (Harrington et al., 2022).

Further, obstructive sleep apnea is a sleep disorder characterized by recurrent episodes of either complete or partial upper airway collapse, leading to episodes of apnea (complete cessation of breathing) or hypopnea (partial reduction of airflow). These respiratory disturbances result in fragmented sleep and daytime drowsiness (Slowik et al., 2023). There is strong evidence suggesting that sleep apnea increases the risk of AD through oxidative stress, intermittent hypoxia, and cardiovascular comorbidities (Andrade et al., 2018). While sleep apnea can occur at any age, the likelihood of its occurrence increases with age and, for women, the risk for sleep apnea drastically increases in the postmenopausal period (Geer & Hilbert, 2021; Rose & Lorenz, 2010).

Social interaction is another factor that contributes to healthy aging. Regular social interactions promote mental health by improving the ability to recover from stress, depression, and anxiety (De Main et al., 2023) as well as promoting physical well-being through immune functioning and decreased inflammation (Geer & Hilbert, 2021; Leschak & Eisenberger, 2019). Further, prolonged social isolation has been associated with decreased cognitive functioning (Lara et al., 2019), and is often associated with loneliness.

Loneliness may also be a key lifestyle factor for AD, given evidence of its association with declines in global cognition, semantic memory, visuospatial ability, and perceptual speed. One study found that older adults experiencing loneliness were 2.1 times more likely to develop AD (Livingston et al., 2020; Mushtaq et al., 2014). Importantly, older adults are at a higher risk of experiencing loneliness compared to their younger counterparts. Recent reports found that 37% of older adults felt a lack of companionship and 33% reported infrequent contact with individuals outside of their home (“Trends on Loneliness,” 2023). Loneliness has also been shown to have differential effects on cognition in men versus women; although women report more loneliness, loneliness was only associated with worse cognitive outcomes in men (Zhou et al., 2019). These findings suggest that studies that account for these sex differences when examining loneliness can be more informative. While it has been shown that sleep quality and loneliness are interrelated (Gyasi et al., 2022), change with age, and are related to cognitive health (McLay et al., 2021), it remains unclear how these risk factors relate to each to each other in individuals at high risk for AD. Understanding the relationship between loneliness, sleep quality, and sleep apnea is important for developing combined intervention methods to simultaneously address multiple risk factors. In the Women: Inflammation Tau Study (WITS), we examined the relationship between the modifiable risk factors of sleep quality, sleep apnea, and loneliness in older women with elevated AD risk, using the Pittsburgh Sleep Quality Index (PSQI) to measure self-reported sleep quality, home sleep tests to measure sleep apnea, and UCLA questionnaire to measure loneliness levels. We hypothesized that loneliness is associated with poor sleep quality and sleep apnea in older women at risk of developing AD.

## Methods Participants

Data were collected from 39 women with the mean age of 72.6 (SD = 4.0) as part of WITS at the University of California, San Diego (UCSD). All participants completed the UCLA Loneliness Scale and PSQI questionnaires and ApneaLink Home Sleep Test for one night. Inclusion criteria for WITS included female sex at birth, at least 65 years of age, and at higher risk for AD by way of an AD polygenic hazard score (Desikan et al., 2017) in the upper 50th percentile and a score on the Telephone Montreal Cognitive Assessment (tMoCA) suggestive of mild cognitive impairment (score range of 13–20 out of 22). Exclusion criteria included: (a) contraindication to lumbar puncture; (b) chronic major psychiatric disorders; major depression by DSM-IV criteria; (c) unstable or poorly controlled medical problems, e.g., heart failure, diabetes (poorly controlled or on insulin), hypertension, a pulmonary disease with hypoxia or hypercapnia, significant liver problems or renal failure, treatment of cancer in the past 2 years, HIV positive; (d) self-reported current substance use disorders; (e) receiving medication in an investigational drug study; (f) use of medications known to impact the CNS in the 4 weeks prior to study visit: neuroleptics, anti-Parkinson's disease medications, CNS stimulants, anticonvulsants, insulin, coumadin, sedating antihistamines or hypnotics, potent antiinflammatory medications, anti-HIV medications; (g) MRI contraindications; (h) major inflammatory disorders, e.g., rheumatoid arthritis, lupus, multiple sclerosis. The research received approval from the Institutional Review Board (IRB) at the University of California, San Diego, under protocol number 200383. All participants provided written informed consent to participate in this study.

### Loneliness Evaluation

Participants completed the UCLA Loneliness Scale, a 20-item questionnaire used to rate perceived feelings of loneliness and isolation. Each item is rated as either O, S, R, or N. O indicates “I often feel this way” and is worth 3 points, S indicates “I sometimes feel this way” and is worth 2 points, R indicates “I rarely feel this way” and is worth 1 point, and N indicates “I never feel this way” and is worth 0 points. A total score is summed from all items (range: 0–60), with higher scores indicating higher levels of subjective feelings of loneliness (Russell et al., 1978).

### Sleep Quality Evaluation

Self-reported sleep quality and disturbance were assessed with the PSQI. This questionnaire has 19 items that measure 7 components of sleep health: (a) sleep quality, (b) sleep latency (how long it takes one to fall asleep), (c) sleep duration (how many hours of sleep one gets), (d) sleep efficiency (the percentage of time spent sleeping while in bed), (e) sleep disturbances (waking up during the night), (f) use of sleep medication, and (g) daytime dysfunction (how does sleep affect daytime functioning) (Buysse et al., 1989). Each item consists of either Likert-type responses or free responses, which are then grouped, scaled, and calculated into 7 component scores (range: 0–3), with specific guidelines for each one (see Buysse et al., 1989). The sleep quality global score is calculated by summing the seven scaled component scores (range: 0–21), with higher scores reflecting poorer sleep quality (Buysse et al., 1989). Participants with sleep quality global scores over 5 were considered “poor sleepers,” whereas participants with sleep quality global scores <=5 were considered “good sleepers” (Buysse et al., 1989).

### Home Sleep Test

Assessment of sleep apnea was done with a home sleep test for one night using ResMed's ApneaLink Air™. The participants wore a respiratory belt, which tracked respiratory effort, a nasal cannula, which detected snores and respiratory flow, and a pulse oximeter, which detected the pulse and blood oxygen saturation levels (“ResMed APneaLink Air Review,” 2023). Diagnosis of sleep apnea followed the American Academy of Sleep Medicine criteria, with an apnea–hypopnea index (AHI) ≥ 5/hr with 3% oxygen desaturations (Kapur et al., n.d.). The presence versus absence of sleep apnea and the AHI were the outcomes of interest.

### Statistical Analyses

ANCOVAs were first conducted to test for differences in total loneliness score between “good sleepers” and “poor sleepers,” as determined by global sleep quality score, and between sleep apnea and non-sleep apnea groups as determined by apnea-hypopnea index while controlling for age as a covariate. For significant differences, we conducted specific analyses of measures comprising each global sleep measure (i.e., sleep quality or subjective, and AHI for objective). For sleep quality components comprised of more than one categorical item (i.e., Subjective Sleep Quality, Sleep Disturbances, Sleep Medications, and Daytime Disturbances), the scaled component scores were examined. Scaled component scores were dichotomized into a score of zero (i.e., that problem did not occur in the past month) and one to three (i.e., that problem occurred at least once during the past month). Then, ANCOVAs with age as a covariate were performed to assess differences in mean total loneliness score between groups. For sleep quality components comprised of a single item that was measured on a continuous scale (i.e., Sleep Latency, Sleep Duration, and Sleep Efficiency), the continuous score of these items were examined. For these three components, linear regression models were performed to examine the relationship between sleep quality measures and total loneliness score, while controlling for age. All statistical analyses were performed in R (version 2023.06.1).

## Results

### Participants Characteristics

Preliminary data were available for 39 women with an average age of 72.6 years (Table 1). Total loneliness scores for our sample ranged from 0 to 31 (*M* = 9.3, SD = 9.5). Sleep quality index global scores ranged from 1 to 13 (*M* = 4.8, SD = 2.9). Thirteen of 39 women (35.14%) were poor sleepers (Sleep Quality Intex Global Score > 5). Among all women with available sleep quality data, the average for habitual sleep efficiency was 88% (SD = 12.2), sleep duration ranged from 5.0 to 10.0 hours (*M* = 7.2, SD 1.2), and sleep latency ranged from 2 to 60 minutes (*M* = 15.7, SD = 13.0). Twenty-three of the 33 women (69.70%) who completed the home sleep test met the criteria for sleep apnea.

**Table 1.** Descriptive Characteristics of Sample.

	Outcome	*n*
Age, *M* (SD)	72.6 (4.0)	39
4-Year College Graduate, %	76.9%	30
Total Loneliness Score, *M* (SD)	9.2 (9.5)	39
PSQI Global Score, *M* (SD)	4.8 (2.9)	37
Poor Sleepers (PSQI Global > 5), %	35.1%	13
PSQI Habitual Sleep Efficiency Percentage, *M* (SD)	88.0 (12.2)	38
PSQI Sleep Duration, *M* (SD)	7.2 (1.2)	38
PSQI Sleep Latency, *M* (SD)	15.7 (13.0)	38
Apnea–Hypopnea Index (AHI), *M* (SD)	12.8 (10.6)	33
Sleep Apnea (AHI ≥ 5), %	69.7%	23

Abbreviations: PSQI, Pittsburgh Sleep Quality Index; AHI, apnea–hypopnea index.

### Associations Between Loneliness and Subjective Sleep Quality

**Figure 1.**
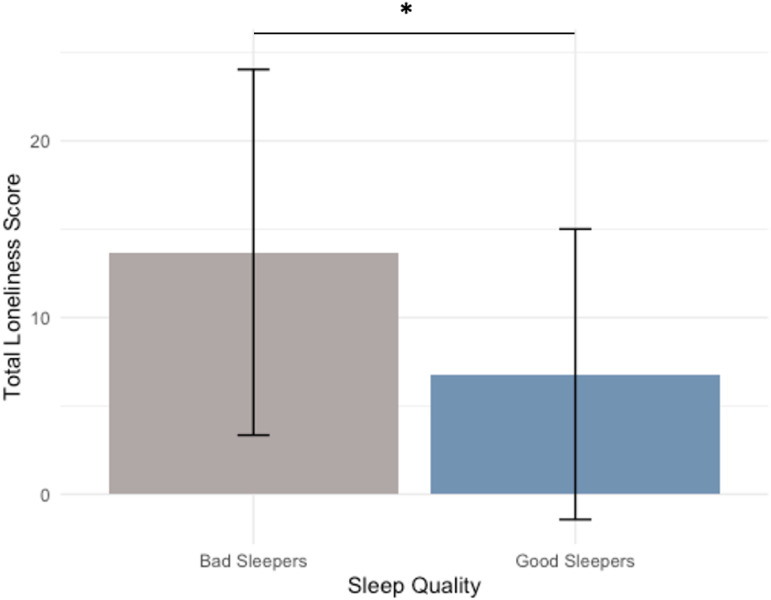
Average total loneliness scores in good vs. poor sleepers.

*Note.* Average total loneliness score in good (*n* = 24) and poor (*n* = 13) sleepers, as determined by global Pittsburgh Sleep Quality Index (PSQI) score. Error bars represent mean for each group ±1 standard deviation. Asterisk represents significance at *p* < .05.

The average total loneliness score was significantly higher in poor sleepers with a mean of 13.7 (SD = 10.3) compared to good sleepers who had a mean loneliness score of 6.8 (SD = 8.2), *F*(1,34) = 4.87, *p* = .03 (Figure 1).

### Associations Between Loneliness and Obstructive Sleep Apnea

**Figure 2.**
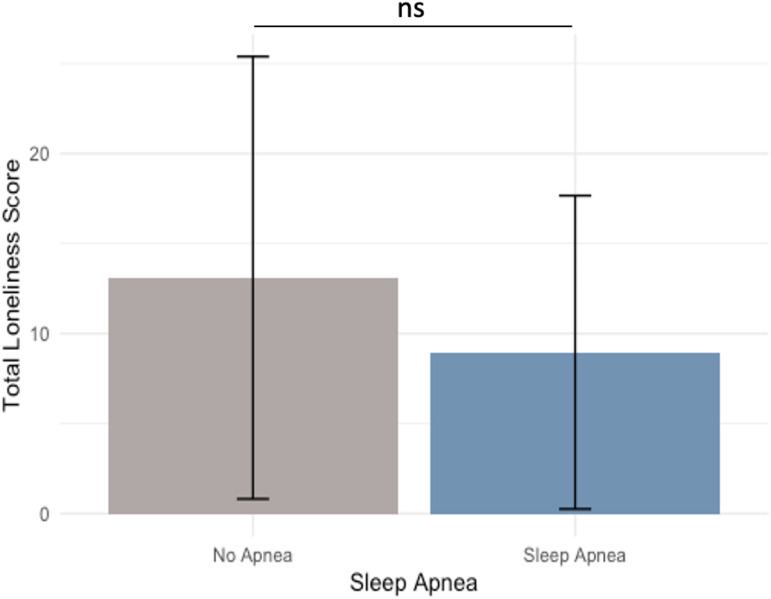
Average total loneliness in apnea vs. non-apnea groups.

*Note.* Average total loneliness scores in apnea (*n* = 23) and non- apnea (*n* = 10) groups, as determined by apnea–hypopnea index (AHI) on a home sleep test. Error bars represent mean for each group ±1 standard deviation. Non-significant difference between groups indicated by “ns.”

There was no significant difference in the average total loneliness scores between those with and without sleep apnea, *F*(1,29) = 0.68, *p* = .42 (Figure 2).

### Associations Between Loneliness and Pittsburgh Sleep Quality Index Components

**Figure 3.**
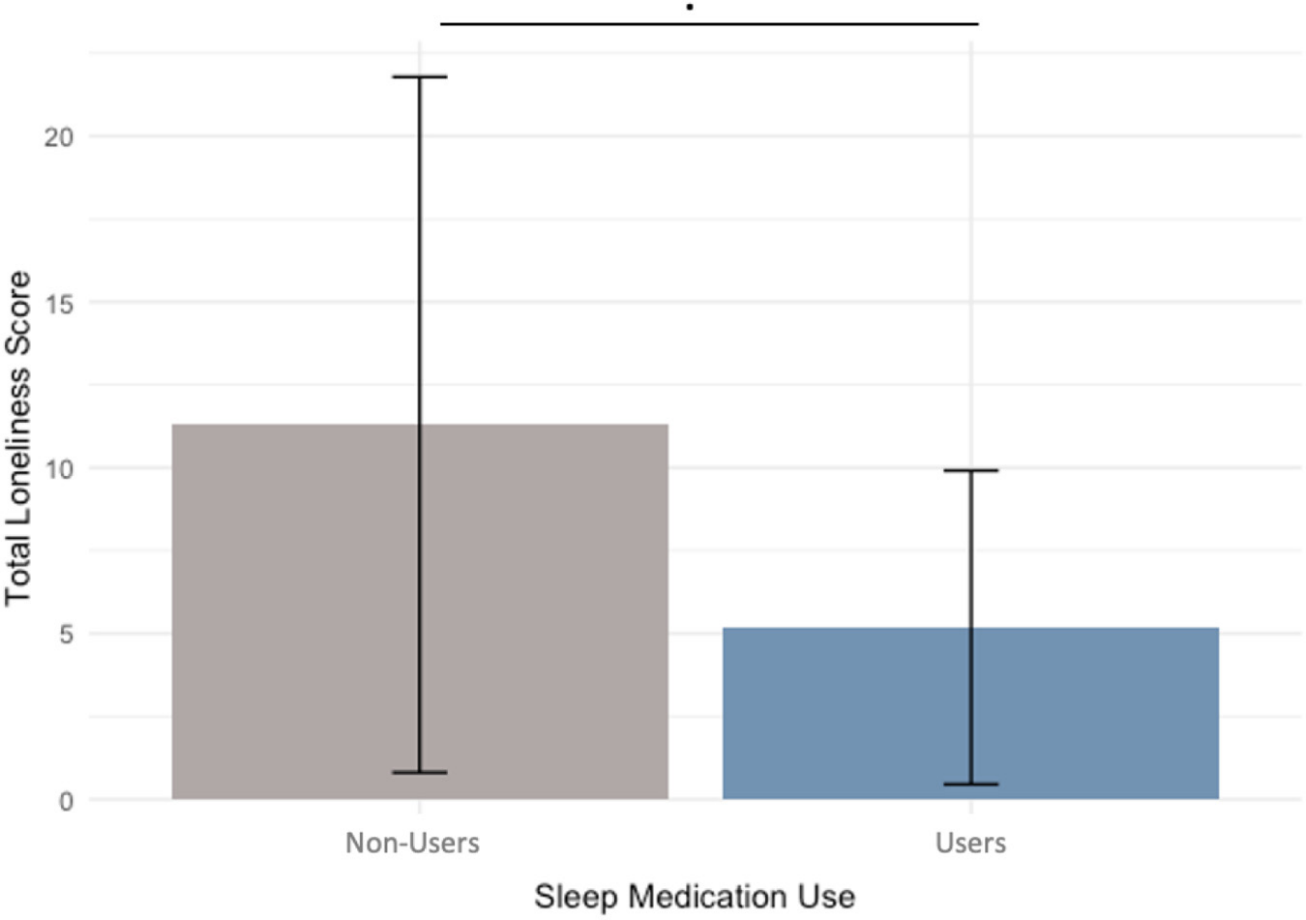
Average total loneliness in sleep medication users vs. non-users.

*Note.* Average total loneliness score in sleep medication users (*n* = 11) and non-users (*n* = 27), as determined by Pittsburgh Sleep Quality Index (PSQI) component 6 score. Error bars represent mean for each group ±1 standard deviation. Dot represents trending significance at *p* < .10.

**Figure 4.**
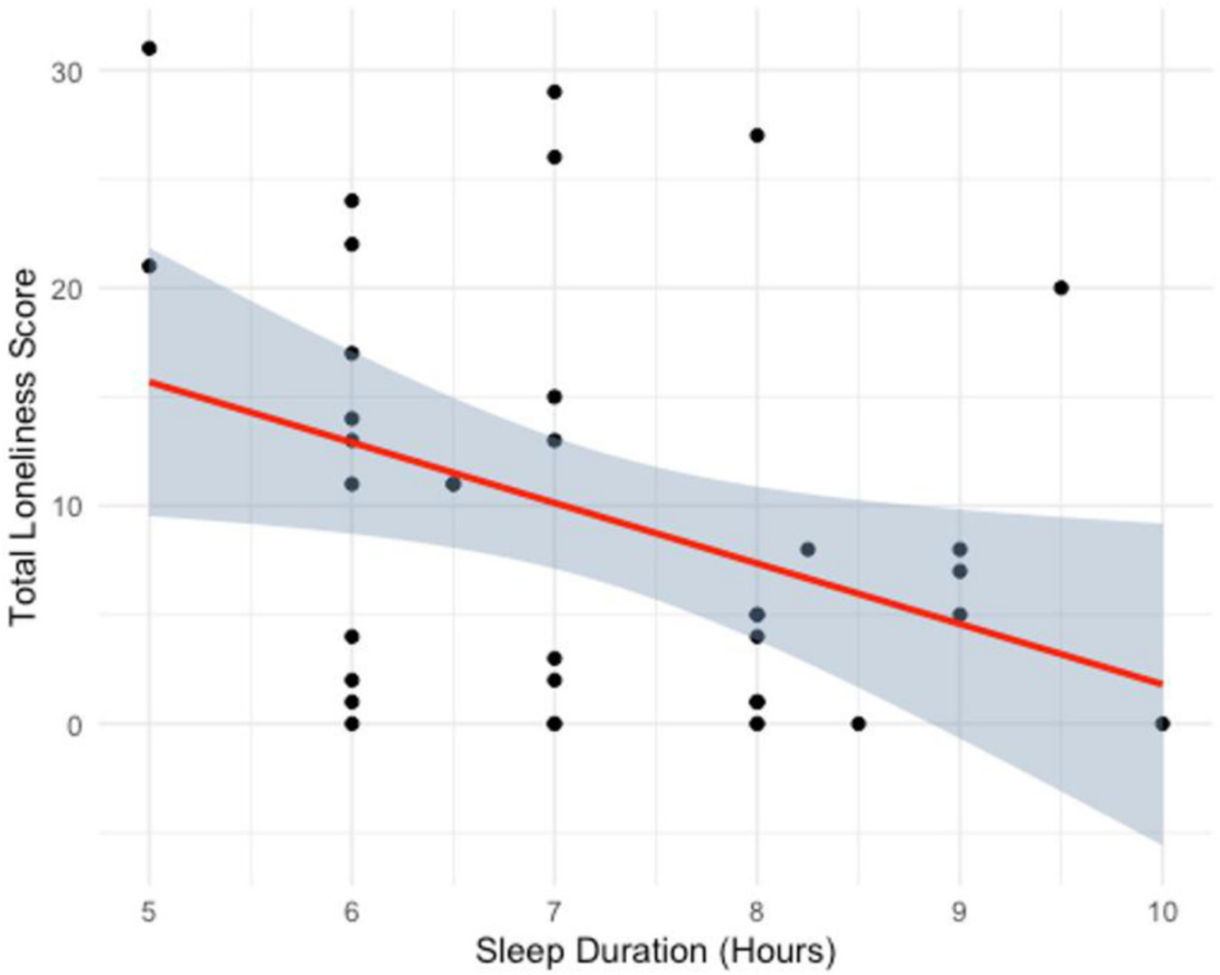
Relationship between total loneliness score and sleep duration.

*Note.* Scatter plot with linear regression line showing relationship between total loneliness score and self-reported sleep duration (hours). The standardized beta coefficient of the linear model when controlling for age is −0.36, *p* = .03. The grey surrounding the red line is the standard error.

**Figure 5.**
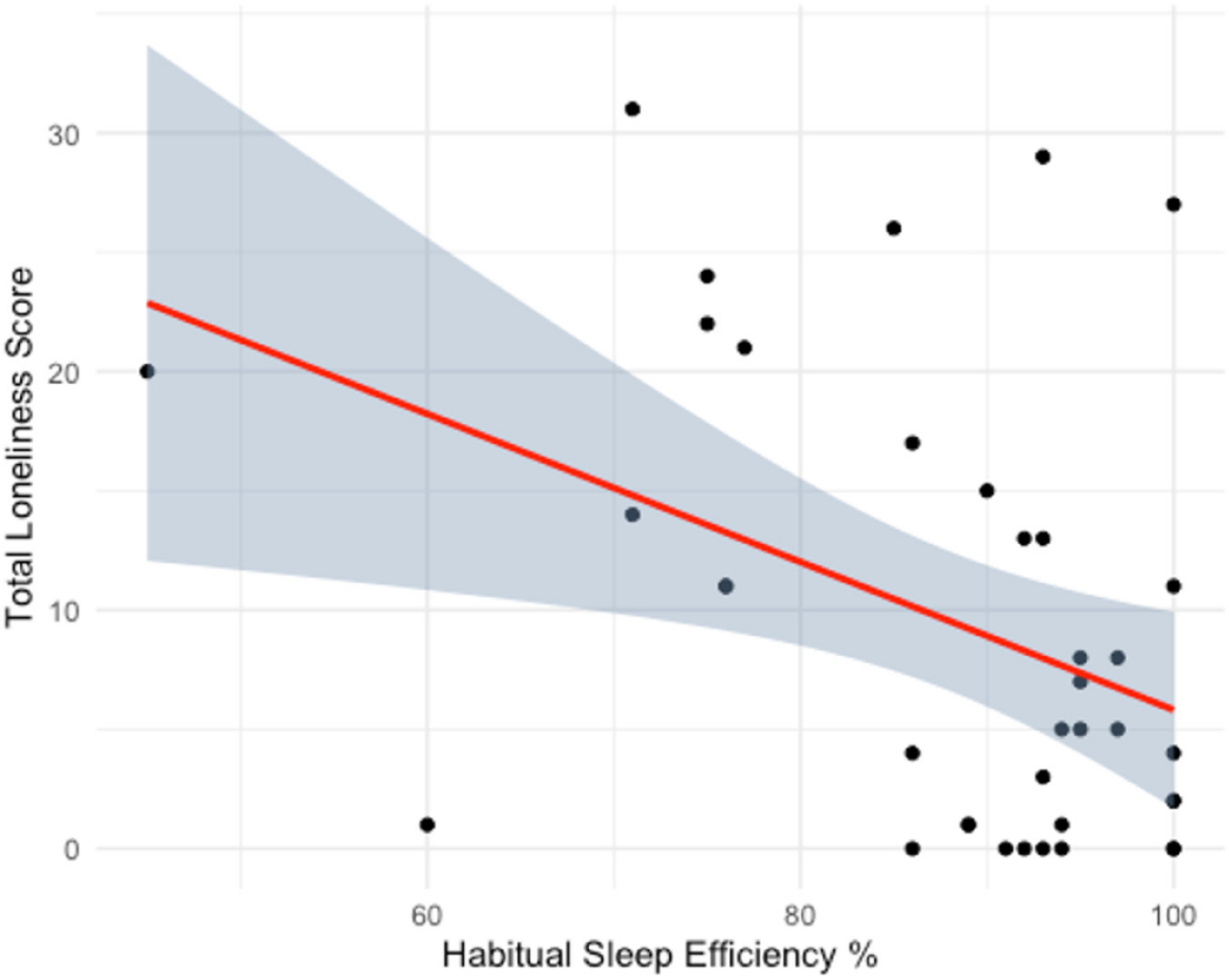
Relationship between total loneliness score and habitula sleep efficiency.

*Note.* Scatter plot with linear regression line showing relationship between total loneliness score and calculated habitual sleep efficiency (percentage of time spent asleep over time spent in bed). The standardized beta coefficient of the linear model when controlling for age is −0.41, *p* = .009. The grey surrounding the red line is the standard error.

We examined whether the significant difference in total loneliness score between good versus bad sleepers was driven by specific sleep quality components. Among Sleep Quality Index components, sleep quality (*F*(1,34) = 1.29, *p* = .27), sleep disturbances (*F*(1,34) = 1.15, *p* = .29), daytime disturbances (*F*(1,34) = 0.66, *p* = .42), and sleep latency (*β* = -0.04, *p* = .83) did not significantly relate to total loneliness scores. Average total loneliness score was trending higher in sleep medication non-users with a mean score of 11.3 (SD = 10.5) compared to users with a mean score of 5.2 (SD = 4.7), *F*(1,34) = 2.91, *p* = .09) (Figure 3). Shorter sleep duration (*β* = -0.36, *p* = .03, Figure 4) and worse habitual sleep efficiency (*β* = -0.41, *p* = .009, Figure 5) were significantly associated with higher total loneliness score.

## Discussion

In this study of older women with increased AD risk, we found marginally significant differences in loneliness between “good sleepers” and “poor sleepers” as defined by the Sleep Quality Index with “poor sleepers” reporting more loneliness. When examining whether specific sleep quality components were driving this difference, we found that shorter sleep duration and lower sleep efficiency were significantly linked to higher loneliness scores, and no sleep medication use was a trending association with heightened loneliness. There was no significant association between sleep apnea and loneliness.

The associations between sleep quality and loneliness are consistent with previous studies that involved both male and female individuals. Notably, one study with 95,045 participants reported that those who experienced social isolation were more likely to report insufficient sleep (McLay et al., 2021). It has been proposed that loneliness can give rise to insomnia symptoms, such as difficulty falling asleep, staying asleep, and early awakening through increased stress, anxiety, and heightened vigilance associated with loneliness (Qi et al., 2023). Despite the well-documented social ramifications of sleep apnea where individuals with sleep apnea tend to engage less in social activities (Jehan et al., 2017), we did not observe a link between sleep apnea and feelings of loneliness.

Loneliness and poor sleep share similar effects on mood, cognition, and overall wellbeing. Lack of social interaction and loneliness can give rise to symptoms of anxiety, depression, and sleep problems, in addition, it has also been linked to AD and autoimmune disorders (Mushtaq et al., 2014). Additionally, most people who experience loneliness report being unhappy and unsatisfied and the shared factors between depression and loneliness has convinced many researchers that loneliness is a subset of depression (Mushtaq et al., 2014). Similarly, poor sleep can increase stress responsivity, cause mood disturbances, bring about cognitive deficits, and affect immune system functions (Medic et al., 2017). Thus, the relationship between poor sleep and loneliness may have implications for the risk of compounding effects of Modifiable Risk Factors on stress, mood disturbances immune health and cognition.

Loneliness and sleep deficiency can have bidirectional effects. As discussed previously, loneliness can give rise to sleep problems by increasing the feelings of anxiety and stress (Owczarek et al., 2022). Similarly, studies have shown that lack of sleep was associated with a behavioral profile of social withdrawal due to sleep deprivation leading to changes in neural mechanisms that increase social repulsion signals (Ben Simon & Walker, 2018). These findings, in combination with our own, suggest that loneliness and disrupted sleep are two risk factors for AD that can have reinforcing effects on each other. Further research is needed to investigate the additive versus synergistic effects of loneliness and poor sleep on AD risk.

There are some limitations to this study that should be addressed. The small sample size limited our statistical power particularly when examining differences between categorical variables with small cell sizes (e.g., good sleepers, no sleep apnea, and sleep medication users) Our sample of predominantly White and well-educated women is not representative of the broader population, limiting the study's generalizability. This study also did not include any objective measures of sleep quality, such as using actigraphy to derive habitual sleep patterns. Relying solely on the reliability of and validity of self-reported data may have introduced potential biases to the results. In light of these limitations, future research with larger and more diverse samples is needed. The integration of objective sleep assessments, such as actigraphy or polysomnography, and additional subjective sleep and loneliness measures, could provide a more comprehensive understanding of the relationship between sleep quality and loneliness. Future longitudinal studies are needed to understand the directionality of our findings, elucidating whether poor sleep quality leads to increased feeling of loneliness or vice versa. Future research may also focus on how mood symptoms such as depression are involved in the relationship between sleep disturbance and loneliness.

The significance of these findings is in their potential to inform how relationships among lifestyle factors such as poor sleep and loneliness can increase the likelihood of a combined effect of multiple lifestyle factors on AD risk. Conversely, we can better understand how intervention approaches targeting a lifestyle factor may have multi-pronged effects that extend beyond a single behavior to improve overall health and well-being. Our finding of a relationship between loneliness and sleep quality combined with prior findings of their association with increased risk of AD, suggests that interventions to improve sleep quality and/or loneliness could be an effective strategy in reducing one's AD risk and improving overall well-being. However, it is important to emphasize that further research with a bigger sample size is needed to validate and expand upon these findings.
